# Effects of dietary substitution of fishmeal by black soldier fly (*Hermetia illucens*) meal on growth performance, whole-body chemical composition, and fatty acid profile of *Pontastacus leptodactylus* juveniles

**DOI:** 10.3389/fphys.2023.1156394

**Published:** 2023-03-27

**Authors:** Maria V. Alvanou, Anastasia Kyriakoudi, Vasiliki Makri, Athanasios Lattos, Konstantinos Feidantsis, Dimitrios K. Papadopoulos, Ioannis Georgoulis, Apostolos P. Apostolidis, Basile Michaelidis, Ioannis Mourtzinos, Adamantia Asimaki, Ioannis T. Karapanagiotidis, Ioannis A. Giantsis

**Affiliations:** ^1^ Department of Animal Science, Faculty of Agricultural Sciences, University of Western Macedonia, Florina, Greece; ^2^ Laboratory of Food Chemistry and Biochemistry, Department of Food Science and Technology, Faculty of Agriculture, Forestry and Natural Environment, Aristotle University of Thessaloniki, Thessaloniki, Greece; ^3^ Laboratory of Animal Physiology, Department of Zoology, School of Biology, Aristotle University of Thessaloniki, Thessaloniki, Greece; ^4^ Laboratory of Ichthyology and Fisheries, Faculty of Agriculture, Aristotle University of Thessaloniki, Thessaloniki, Greece; ^5^ Department of Ichthyology and Aquatic Environment, University of Thessaly, Volos, Greece

**Keywords:** freshwater crayfish, Pontastacus leptodactylus, Hermetia illucens, juveniles, insectmeals PUFA/SFA

## Abstract

Freshwater crayfish are considered as aquatic products of high quality and high nutritional value. The increasing demand has led to populations reduction in several locations throughout their range. Thus, the development of appropriate rearing conditions is considered necessary, among which, optimization of their diet is a basic part. Towards this direction, in the present study, a 98-day feeding trial was carried out to evaluate the impact of dietary fishmeal substitution by *Hermetia illucens* meal on *Pontastacus leptodactylus* juveniles kept under laboratory conditions. Insect meals represent an environmentally friendly alternative solution, considered as a high-value feed source, rich in nutrients such as protein and fat. Three dietary regimens were utilized with a fishmeal-based without *Hermetia* meal (HM) defined as the control diet (HM0), and two diets, the first with 50% (HM50) and the second with 100% (HM100) of fishmeal substitution by HM, respectively. Growth performance, whole-body composition, and fatty acid profiles of individuals were studied in the different treatments. At the end of the feeding trial, statistically significant differences were observed in the mean survival rate (SR), specific growth rate (SGR), feed conversion ratio (FCR) and weight gain (WG) values. More specifically, animals fed with HM-based diets had higher mean SR, while the control group performed better regarding FCR and SGR. The HM inclusion in the diet significantly altered the whole-body chemical composition of the crayfish signifying a different metabolic utilization compared to fishmeal (FM). The fatty acid analysis revealed that 16:0 (palmitic acid) was the predominant saturated fatty acid (SFA), 18:1ω9 (oleic acid) was found to be the main monounsaturated fatty acid (MUFA), while 18:2ω6 (linoleic acid) represented the major polyunsaturated fatty acid (PUFA) followed by C20:3 cis ω3 (cis-11-14-17-eicosatrienoate) and C22:6 cis ω3 (cis-4,7,10,13,16,19-Docosahexaenoic) fatty acids. The inclusion of dietary HM significantly reduced the contents of ∑SFAs, ∑PUFAs and ∑ω6 fatty acids, as well as those of C22:6 cis ω3 and increased the ω6/ω3 and hypocholesterolemic to hypercholesterolemic ratios in the body. In parallel with improvements in balanced diets and in culture conditions that need to be optimised for rearing of freshwater crayfish, our study provides new data that enlighten the suitability of insect meals in the nutrition of *P. leptodactylus*.

## 1 Introduction

Apart from being keystone species and ecosystem scavengers (Usio and Townsend, 2008) freshwater crayfish possess a high economic and cultural value since ancient times (Koutrakis et al., 2009) and nowadays crayfish catches reach high prices (Ackefors, 1998; Jussila et al., 2015). Also, more recently, in Northern Greece crayfish festivals take place in an annual base (Alvanou et al., 2022), reflecting its high economic importance at a local level. Its high value in human diet is not only attributed to the high content and quality of proteins and fats but also to its consumption during fasting periods complying with religious guidelines (Jö, 2004; Patoka et al., 2016).

*Pontastacus leptodactylus* is a native species in northeastern Greece. However, due to its high economical value and its export potential, which led to overfishing, its population in lake Vegoritida and lake Polyphytou in Northern Greece seems to decline (Alvanou et al., 2022). Freshwater crayfish populations’ declines have been observed in several studies (Bolat, 2001; Harlioğlu and Harlioğlu, 2009; Souty-Grosset and Reynolds, 2009) and in most cases were attributed to a combination of overfishing, pollution and habitat loss or destruction (Edsman et al., 2010; Karimpour et al., 2011). Generally, freshwater crayfish poses an essential role among freshwater ecosystems as it is characterized by opportunistic feeding behavior. They can consume algae, macrophytes, other invertebrates, small fish, as well as remains of animal tissues and detritus (Gutiérrez-Yurrita et al., 1998; Gherardi and Barbaresi, 2008; Twardochleb et al., 2013).

As the demand for decapod crustacean fisheries is growing globally (Boenish et al., 2022), it is estimated that the fishing rates will increase even more leading to overexploitation of wild stocks and the devastation of the native populations. Hence, it is of major importance to develop a rearing protocol for both restocking purposes and coverage of global consumption demands for crustaceans (Pantazis et al., 2015; Seemann et al., 2015; Alvanou et al., 2022).

At the same time, the nutritional need for crayfish farming requires large amounts of fishmeal which is the main protein source used in crayfish feeds. However, the limited availability of fishmeals has resulted in increased market price, reaching even double prices in comparison to 10 years ago (Dalsgaard et al., 2009). Keeping this in mind in combination with the environmental cost, the scientific community provides efforts to find alternative protein sources for aquaculture use, as fishmeals are included in 20%–50% in crayfish diets leading to increased feed costs and production (Makkar et al., 2014; Qian et al., 2021). Among them, insect meals represent an important alternative solution mainly due to their high nutritional value (Makkar et al., 2014; Henry et al., 2015; Nogales-Mérida et al., 2018). Apart from their important nutrients, insect meals are considered more environmentally friendly, as they are characterized by lower environmental footprint in comparison to conventional animal and plantfeed ingredients (Van Huis and Oonincx, 2017). More specifically, the black soldier fly (*Hermetia illucens* Linnaeus 1758) is a fly (Order: Diptera) within the Stratiomyidae family. *H. illucens* larvae is considered as a high-value feed source with a high percentage of protein and fat, as it can contain up to 60% crude protein and up to 40% crude lipid (Makkar et al., 2014; Henry et al., 2015; Nogales-Mérida et al., 2018). This insect species is also rich in Ca (5%–8% dry matter) (Newton et al., 1977; Arango et al., 2004; Newton et al., 2005), an element of high importance for crayfish, as it is necessary to build their new exoskeleton after molting (Aiken and Waddy, 1992). Furthermore, *H. illucens* can grow while feeding on waste residues resulting in the actual conversion of waste into high nutritious feed ingredients (Diener et al., 2009).

Most of the published studies using *H. illucen*s meal for fishmeal replacement have been conducted majorly in farmed fish species such as rainbow trout (Stadtlander et al., 2017), Atlantic salmon (Li et al., 2020), European sea bass (Magalhães et al., 2017), gilthead seabream (Karapanagiotidis et al., 2023), African catfish (Fawole et al., 2020), Nile tilapia (Tippayadara et al., 2021) and yellow catfish (Xiao et al., 2018), and only few in crustaceans such as pacific white shrimp (Cummins et al., 2017) and red claw crayfish (Wang et al., 2022). For *P. leptodactylus*, Mazlum et al. (2021) evaluated the use of *Tenebrio molitor* and reported that a dietary inclusion level of 13.5%, representing 50% of fishmeal replacement, was successful in terms of growth performance.

Previous studies revealed no harmful effects of HM insect meals on both digestibility and growth performance of the organisms farmed (Renna et al., 2017; Xiao et al., 2018). However, there are conflicting results regarding the optimal substitution levels. The study conducted on rainbow trout suggested that 40% ΗΜ inclusion, may result at no negative effects on fish physiology or meat quality, however a decrease in desirable polyunsaturated fats was observed (Renna et al., 2017) while a maximum level of 15% inclusion was suggested for unaffected fish growth from a second study (St-Hilaire et al., 2007). Additionally, Wang et al. (2022) concluded that for *Cherax quadricarinatus* juveniles the optimal dietary HM inclusion was determined to be 17.1%. From studies conducted on *Litopenaeus vannamei*, it was found that 30% HM inclusion negatively affected the growth performance and body composition of the animals (Chen et al., 2022) while the best growth performance was achieved at a substitution amount of 15% (Hu et al., 2019). In *P. leptodactylus* juveniles, it was revealed that the addition of *T. molitor* was promising regarding growth performance, suggesting that the optimal substitution level of fishmeal with the insect meal was 50% (Mazlum et al., 2021).

A few studies have been conducted to estimate the optimal dietary requirements of crude proteins and fat of *P. leptodactylus* (Ackefors et al., 1992; Carral et al., 2011; Ghiasvand et al., 2012; Valipour et al., 2012). Furthermore, many dietary supplements have been added to *P. leptodactylus* meals for survival and growth performance, body composition, immunity, and stress resistance assessment (Zahmatkesh et al., 2005; Mazlum et al., 2011; Valipour et al., 2011; Harlıoğlu et al., 2014; Bahadir Koca et al., 2015; Safari et al., 2015; Sirin and Mazlum, 2017; Nedaei et al., 2019; Jalili et al., 2020; Mazlum and Şirin, 2020; Safari et al., 2021). Apart from growth performance, there are also some dietary factors that affect the efficiency towards reproduction of the species (Harlıoğlu and Farhadi, 2017). More specifically, phospholipids, dietary lipids, unsaturated fatty acids, vitamins, carotenoids, proteins, and amino acids, seem to play a crucial role towards broodstock crayfish reproduction (Harlıoğlu and Farhadi, 2017). From all the above studies (Zahmatkesh et al., 2005; Mazlum et al., 2011; Valipour et al., 2011; Harlıoğlu et al., 2014; Bahadir Koca et al., 2015; Safari et al., 2015; Harlıoğlu and Farhadi, 2017; Sirin and Mazlum, 2017; Nedaei et al., 2019; Jalili et al., 2020; Mazlum and Şirin, 2020; Safari et al., 2021), it has been implied that the recommended dietary protein level ranges between 30%-39% while diet with higher protein percentage apart from having higher cost has nothing to offer to overall crayfish performance. Concerning lipid content, the optimal growth has been observed in a diet lipid content among 10%–13%. Additionally, many by-products and waste ingredients had been added to crayfish diets such as olive mill wastewater (Parrillo et al., 2017), pikeperch faeces (Roessler et al., 2020) and shrimp waste meal (Bahadir Koca et al., 2011) exhibiting ambiguous results on growth and health of crayfish. More specifically, although olive mill wastewater and 10% shrimp waste mill substitution had positive effects on final weight and weight gain, pikeperch faeces failed to increase the growth performance. However, apart from the investigation of a nutrient balanced diet, the suitability of protein sources in the diet is of high importance as well. Fishmeal is considered the major protein source in aquaculture, but insects are gaining increased attention due to their high nutritional value (Makkar et al., 2014; Henry et al., 2015; Nogales-Mérida et al., 2018), their low environmental footprint (Van Huis and Oonincx, 2017) and their beneficial potential towards immune system (Mousavi et al., 2020).

Therefore, the aim of the study was to assess the effects of fishmeal replacement by HM. Although other researchers studied insect meal substitutions, the results regarding crustaceans are limited and contradictory. Hence, the main scope of the present study was to investigate the growth performance, the survival rate, and the whole-body chemical composition of *P. leptodactylus* juveniles after the replacement of fishmeal with *H. illuciens* worm meal in their diet, as a necessary step towards the optimization of freshwater crayfish rearing protocol. Furthermore, the fatty acid profile was analyzed and the effect of the different dietary sediments on ∑SFAs, ∑PUFAs, ∑MUFAs, ∑ω3 and ∑ω6 fatty acids was evaluated.

## 2 Materials and methods

### 2.1 Origin and collection of experimental animals

**FIGURE 1:**
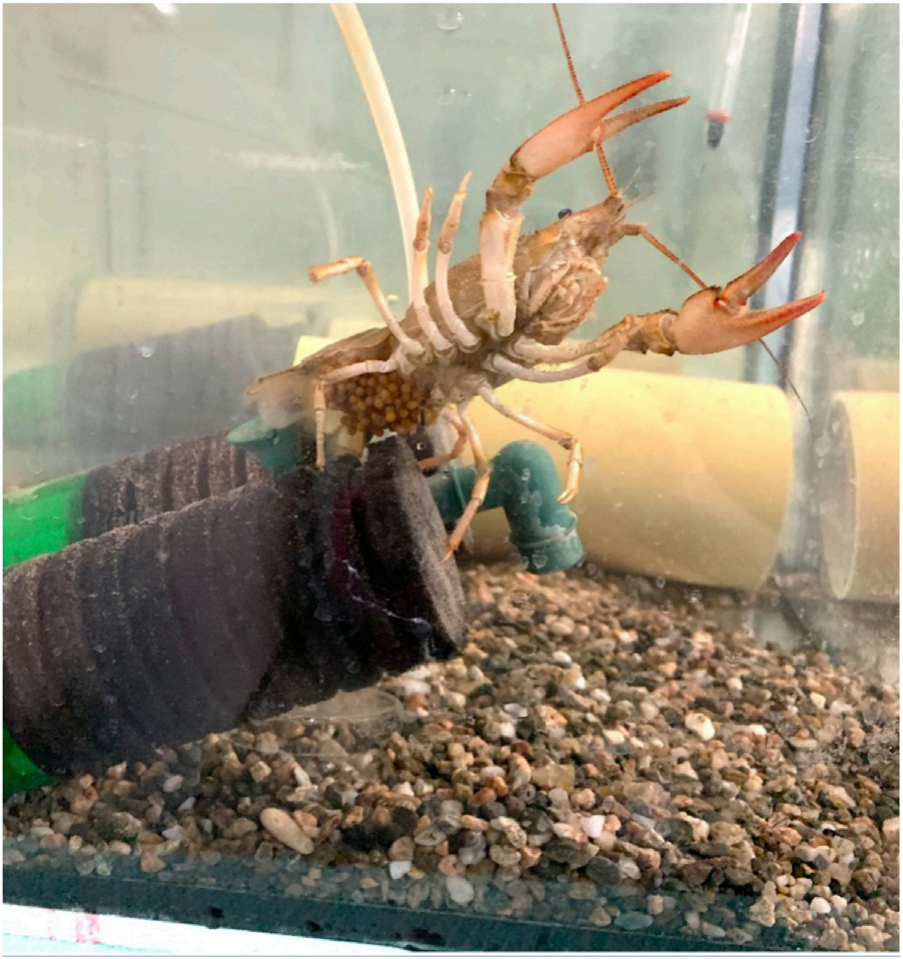
Ovigerous *P. leptodactylus* crayfish collected from the lake Vegoritida and transferred to laboratory conditions.

**FIGURE 2:**
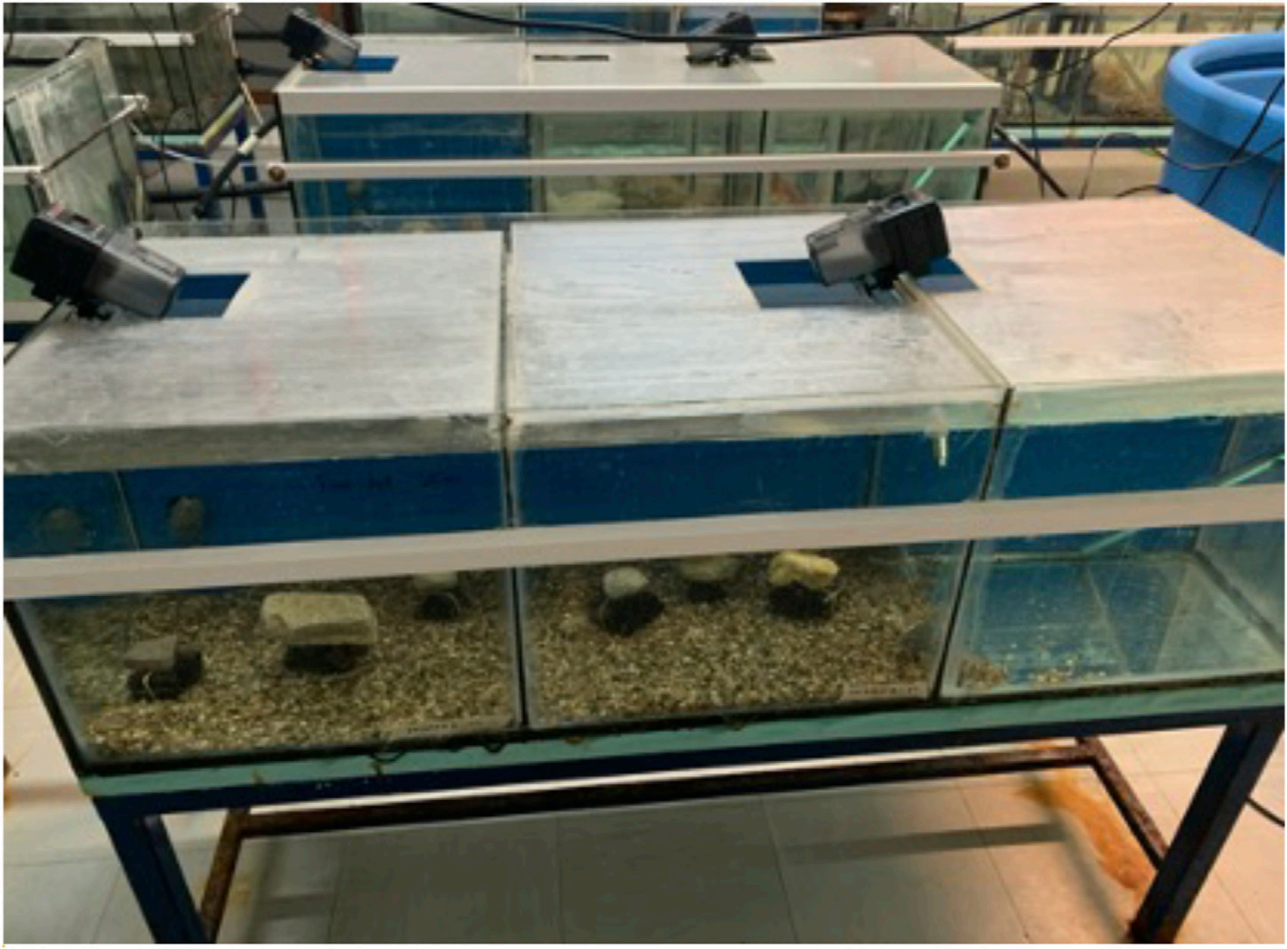
Aquaria (40 × 45 × 50, 90 L) where *P. leptodactylus* juveniles were reared.

Narrow-clawed crayfish (*P. leptodactylus*) individuals with eggs (Figure 1) were collected from the lake Vegoritida located in the borders of Florina and Pella Regional Units, Macedonia, north Greece. On 21st of February 2022, the four collected specimens were transferred to the laboratory of Animal physiology of the School of Biology of Aristotle University of Thessaloniki, located in Thessaloniki, Greece. The ovigerous crayfish were placed in aquaria of 70 L capacity (40 cm long, 50 cm width, 35 cm height) all equipped both with air pumps and PVC shelters (Figures 1, 2). The water temperature was 17 ± 0.72°C (Farhadi and Jensen, 2016), while a photoperiod 12:12 (Light: Dark) (Farhadi and Harlıoglu, 2018) was applied. Each individual kept separately in each aquarium, so the stocking density was 1 individual/0.2 m^2^. Female individuals were fed a commercial feed (crude protein 45%, crude lipid 17%) for *Sparus aurata* to develop and release eggs. The eggs from the 4 ovigerous crayfish hatched on seventh of April. After hatching the juveniles were grown until the first of July and used for the needs of the present study.

### 2.2 Experimental protocol

**FIGURE 3:**
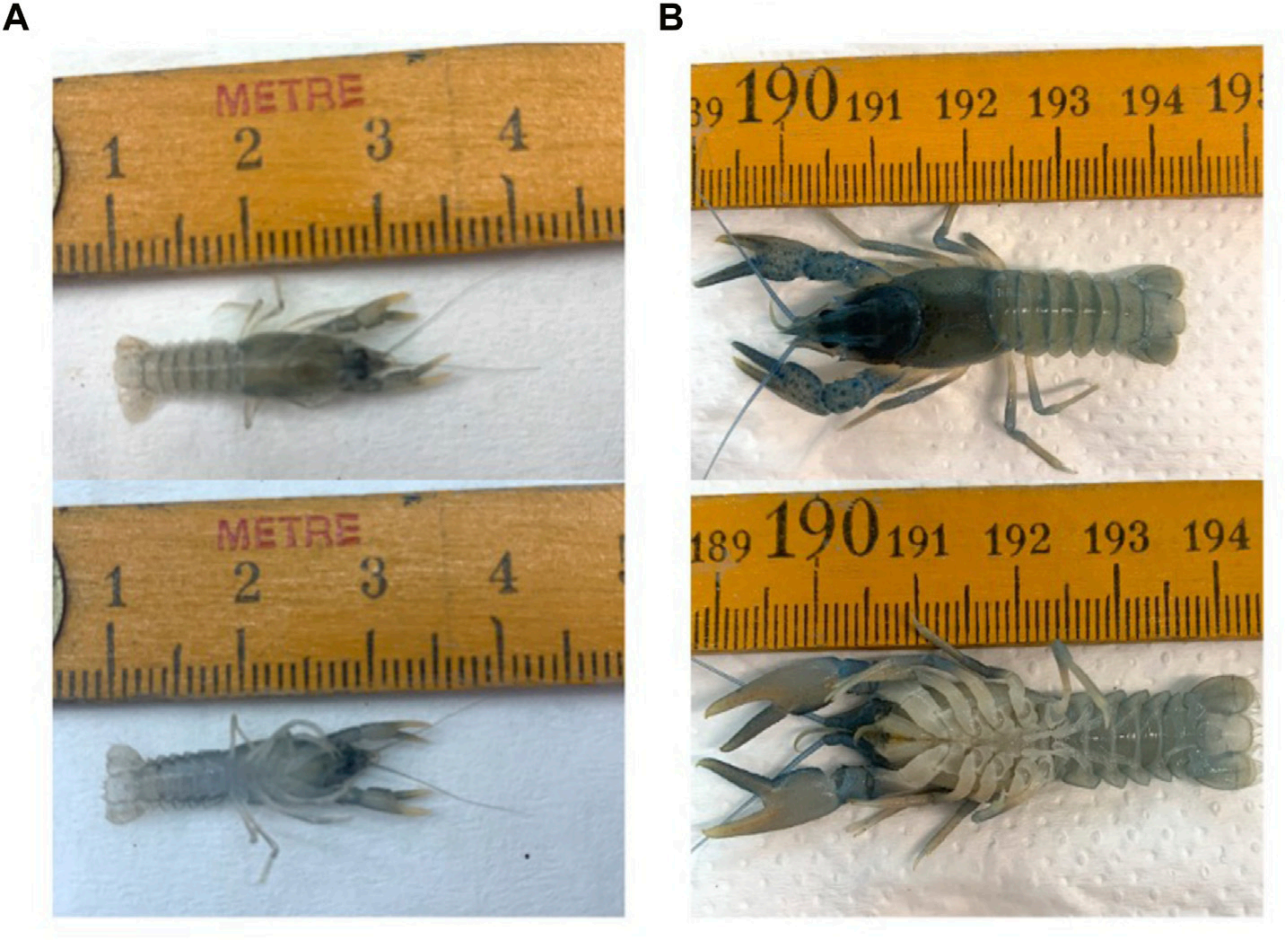
*P. leptodactylus* before **(A)** and after **(B)** the 98-days feeding trial.

The experiment was conducted in six independent aquaria (90 L, 40 cm long, 50 cm width, 45 cm height) (Figure 2). The aquaria were equipped with continuous aeration system to maintain high oxygen levels and heaters to keep constant water temperature at 20 ± 0.5°C. A 30% water exchange was practiced daily in each tank, while a photoperiod 12:12 (Light: Dark) was applied. *P. leptodactylus* juveniles with an average initial body weight of 0.26 ± 0.04 g (Figure 3) were randomly stocked into the six aquaria (two replicate aquaria per dietary treatment with 20 crayfish per aquarium). The feed was supplied two times daily at 11:00 and 19:00 by hand and by mechanical feeders at 5% of total biomass for 98 days. Temperature, pH, conductivity, dissolved oxygen, and mortality were recorded daily with mean values of pH 8.2 ± 0.03, dissolved oxygen 7.4 ± 0.23 mg/L, salinity 656 ± 100 μS. Dead individuals were recorded daily and removed. Any uneaten feed was siphoned the next morning and then was filtered, dried and weighted to calculate the amount of food that was consumed.

### 2.3 Experimental diets

Larvae of *H. illucens* were reared in the University of Thessaly on a mixture of wheat bran (90%) and egg layer hens feed (10%). Late-instar larvae were collected, dried at 40°C for 12 h, milled and partially defatted using petroleum ether at 40°C for 3 h in order to produce a *H. illucens* meal (HM) containing 10.3% moisture, 48.5% crude protein, 10.0% crude lipid, 9.3% ash and 19.9 KJ/g gross energy.

A fishmeal of 64.1% crude protein was used and included at 25% in the control diet. Three isonitrogenous (40% as fed), isolipidic (16%) and isoenergetic (22 MJ/Kg) diets were formulated (Table 1), where the fishmeal protein of the control diet was replaced by HM at 50% (H50) and 100% (H100), respectively. The HM diets were supplemented by methionine to counterbalance their lower levels in this amino acid compared to the HM0 control diet. In all diets, soybean concentrate, sunflower and corn gluten were used as the major plant protein sources, while wheat meal was used as the binder and filler ingredient for the protein replacements. Fish oil was used as the major lipid source and as a source of ω3 fatty acids, while soybean oil was used as an extra lipid and energy source and to counterbalance the isolipidic diets. All the diets had constant inclusion levels of vitamins and minerals premix, monocalcium phosphate and vitamin C (Table 2).

**TABLE 1:** Proximate composition (% of wet weight) of the partially defatted *H. illucens* meal and of fishmeal used in the experimental diets.

	*H. illucens* meal^a^	Fishmeal^b^
Moisture (%)	10.3 ± 0.3	7.8 ± 0.1
Crude protein (%)	48.5 ± 0.4	64.1 ± 0.3
Crude lipid (%)	10.0 ± 0.6	8.7 ± 0.2
Ash (%)	9.3 ± 0.4	16.1 ± 0.1
Gross energy (KJ/g)	19.9 ± 0.2	20.5 ± 0.2

Values represent means ± standard deviation (n = 3).

^a^ *H. illucens* defatted meal produced by the University of Thessaly.

^b^ Fishmeal, sardine Köster Marine Proteins GmbH, Hamburg, Germany.

**TABLE 2:** Formulation (g/kg of diet), proximate composition (% in dry matter) and estimated amino acid content (% of protein) of the experimental diets.

Diets	HM0	HM50	HM100
*Ingredients (%)*			
Fishmeal	250	125	—
*Hermetia illucens* meal	—	165.4	330.9
Soybean concentrate	180	186	192.1
Sunflower	100	100	100
Corn gluten	100	100	100
Wheat bran	180	139.6	98
Fish oil	70	70	70
Soybean oil	45	38	32
Methionine	—	1	2
Vitamin and mineral, premix^a^	10	10	10
Monocalcium phosphate	60	60	60
Vitamin C	5	5	5
*Proximate composition (% in DM)*			
Crude proteins	44.5	44.4	44.5
Crude lipids	16.2	15.8	15.8
Crude carbohydrates^b^	26.0	26.9	27.3
Ash	13.5	12.9	12.4
Gross energy (KJ/g)	22.3	22.2	22.2
*Estimated amino acids (% of protein)* ^c^			
Alanine	3.89	4.27	4.65
Arginine	4.00	4.06	4.10
Aspartic acid	5.74	6.28	6.80
Cysteine	1.05	0.88	0.69
Glutamic acid	13.40	12.58	11.65
Glycine	3.53	3.58	3.61
Histidine	1.64	1.77	1.89
Isoleucine	2.89	3.08	3.26
Leucine	5.76	5.94	6.09
Lysine	3.73	3.78	3.82
Methionine	1.47	1.48	1.48
Phenylalanine	3.29	3.50	3.68
Proline	4.51	4.76	4.97
Serine	3.17	3.07	2.94
Threonine	2.63	2.63	2.62
Tryptophane	0.79	0.69	0.59
Tyrosine	1.55	2.21	2.86
Valine	3.45	4.02	4.57

^a^ Vitamin and mineral premix (per kg of mixture): vitamins: E, 58.3 g; K3, 3.3 g; A, 1,500 IU/g; D3, 200 IU/g; B1, 3.3 g; B2, 6.6 g; B6, 3.3 mg; B12, 10 mg; folic acid, 3.3 g; biotin, 100 mg; inositol, 40 g; C, 33.3 g; nicotinic acid, 16.6 g; pantothenic acid, 13.3 g. Minerals: Co., 170 mg; I, 248 mg (Ca(IO3)2); Mn, 10 g (MnO); Zn, 33 g (ZnO); Ca 235 g; Se 2.5 mg (Na2SeO3); Na 247,5 mg (Na2SeO3); Fe, 2 g; Mg, 121,3; Cu, 0.8 g.

^b^ Calculated as 100 minus the sum of the percentages of crude protein, crude fat, moisture and ash.

^c^ Based on the amino acid profiles of each ingredient given by the suppliers, while for *H. illucens* meal the average value of each amino acid from feedipedia.org were used.

Diets were prepared in the University of Thessaly, Greece. All dietary ingredients were ground in a grain feed mill (KoMo Fidibus, PGS, Germany) and were mixed in a mixer (Bosch MaxxiMUM MUMXL20G). The oils and boiling water were then added to produce a homogenous stiff dough. Diets were pelletized by a California Pellet Mill (CL-2, IRMECO GmbH, Netherlands) to produce pellets of 2.5 mm diameter. The pellets were then dried with forced air at room temperature for 24 h to reach a moisture content of 8%–8.5% and then stored in air-sealed bags at 4°C until used.

### 2.4 Growth performance and feed utilization

At the end of the 98-days feeding experiment all live individuals were collected after 48 h fasting. After collection, each individual was dried carefully with a tissue paper to remove the excess water and then weighted. Apart from weight, other parameters that were measured included carapace, chelae, and abdomen lengths and widths.

The following parameters were calculated for growth performance and feed utilization:

• Survival rate (SR, %) = (Total number of crayfish harvested/Total number of crayfish stocked) × 100
• Specific growth rate (SGR, %/day) = [In Final body weight- In initial body weight/rearing duration (days)] × 100
• Weight Gain (WG, g) = final weight (g)—initial weight (g)
• Feed Conversion Ratio (FCR) = Dry feed intake (g)/wet weight gain (g)
• Protein efficiency ratio (PER) = weight gain (g)/protein intake (g)

### 2.5 Whole body composition including exoskeleton

Samples were homogenized using liquid nitrogen and then were freeze-dried using a HyperCOOL HC8080 freeze-dryer (Gyrozen Co., LTD., Korea) (−80°C, 0.1 mbar). Moisture content was determined by oven drying at 105°C for 24 h, crude protein content (N x 6.25) by the Kjeldahl method using a Gerhardt analytical apparatus (Association of Official Analytical Chemists–AOAC, 2000), and crude ash content by incineration at 550°C for 5 h using a muffle furnace (L 9/11/B180 L-090H1CN, Nabertherm GmbH, Lilienthal/Bremen, Germany). Total lipids were extracted using the Folch method (Folch et al., 1957). In particular, 1 g of each freeze-dried and ground crayfish sample was mixed with 20 mL of a solution of chloroform: methanol (2:1, v/v) and were vigorously agitated for 45 min. The extraction was repeated twice. After filtering, water was added for the phase separation. The upper phase was removed and the lower chloroform was collected, dehydrated with anhydrous Na_2_SO_4_ and rotary-evaporated to dryness.

### 2.6 Determination of total lipids and fatty acids profile

After total lipids extraction that was carried out as described above, transesterification was carried out to the samples for subsequent gas chromatographic analysis. In particular, 0.1 g of the extracted lipids was transferred in a test tube with a screw cap and 2 mL of n-hexane were added, followed by 0.2 mL of a 2 M methanolic solution of potassium hydroxide for the fatty acid methyl esters (FAMEs) preparation. The mixture was vortexed for 1 min and was left to settle until the upper phase that contained the FAMEs became transparent. The phase that contained the methyl esters was collected, filtered (0.45 µm PTFE hydrophobic filters) and analyzed by a gas chromatograph (TRACE GC 2000 Series, Thermo Quest CE Instruments) with a flame ionization detector (FID) equipped with an autosampler (TRIPLUS AS Thermo Quest CE Instruments). FAMEs were analyzed on a BPX70 GC column (30 m length, 0.32 mm i.d., 0.25 μm film thickness, SGE Analytical Science). Helium was the carrier gas at a flow rate of 2.0 mL/min. The injector port and detector temperature were maintained at 250°C. The split ratio was 1:20. The column oven was initially set at 46°C for 2 min, then increased to 130°C at a rate of 50°C/min for 10 min, then increased to 175°C at 2°C/min and maintained at that temperature for 2 min, then increased to 200°C at 3°C/min and maintained at that temperature for 3.5 min, before increasing to a plateau of 240°C at a rate of 10°C/min for 5 min. The total run time was 60 min. The identification of FAMEs was carried out by comparing the retention times (RT) with those of a standard mixture (AccuStandard, New Haven, United States) containing 37 fatty acids analyzed under the same chromatographic conditions. Chromatograms were acquired and processed with the aid of Chrom Quest 5.0 software (ver. 3.2.1, Thermo Separation Products).

Parameters useful for evaluating the nutritional value of fats were also determined. In particular, the sum of the saturated fatty acids (∑SFA), monounsaturated fatty acids (∑MUFA), polyunsaturated fatty acids (∑PUFA), ω3 fatty acids (∑ω3) and ω6 fatty acids (∑ω6), as well as the ratios of ∑PUFA to ∑SFA (∑PUFA/∑SFA), ∑ω6 to ∑*ω*3 fatty acids (∑*ω*3/∑*ω*6) and hypocholesterolemic to hypercholesterolemic (H/H) fatty acid ratio. The H/H ratio was determined as follows: H/H = sum (∑) of 18:2ω9, 18 ω6, 20:4ω6, 18:3ω3, 20:3ω6, 20:5ω3, 22:6ω3/sum (∑) of 14:0, 16:0 (Chen and Liu, 2020).

### 2.7 Statistical analysis

Parameters examined herein were tested for significance at the 5% level (*p* < 0.05) by using one way (GraphPad Instat 3.0) analysis of variance (ANOVA). Values are presented as means ± S.D. Friedman’s non-parametric test, followed by Dunn’s post-test, was performed to re-analyse and cross-examine our data. Post-hoc comparisons were performed using Bonferroni test. Principal components analysis (PCA) in the FactoMineR package in R was employed to assess patterns of possibly correlated variables, and more specifically to detect how fatty acids’ levels varied between treatments.

## 3 Results

### 3.1 Survival, growth performance and feed utilization

**FIGURE 4:**
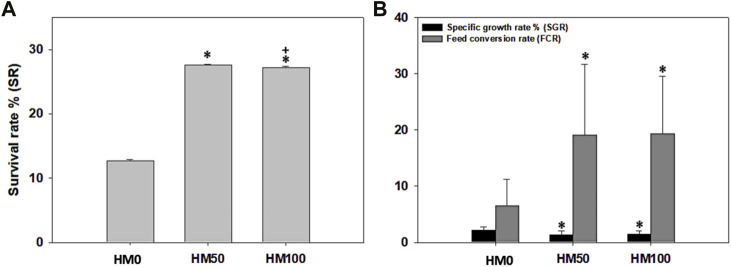
**(A)** Survival rate % (SR), **(B)** specific growth rate % (SGR) and feed conversion rate (FCR) levels (mean ± stedv) between different dietary treatments (HM0, HM50 and HM100) in *P. leptodactylus* juveniles. Asterisk (*) depicts statistically significant differences (*p* < 0.05) between HM treatments and HM0, while cross (+) depicts statistically significant differences (*p* < 0.05) between HM50 and HM100 treatments. HM0 represents the control group while HM50 and HM100 the groups with 50% and 100% HM inclusion respectively.

**FIGURE 5:**
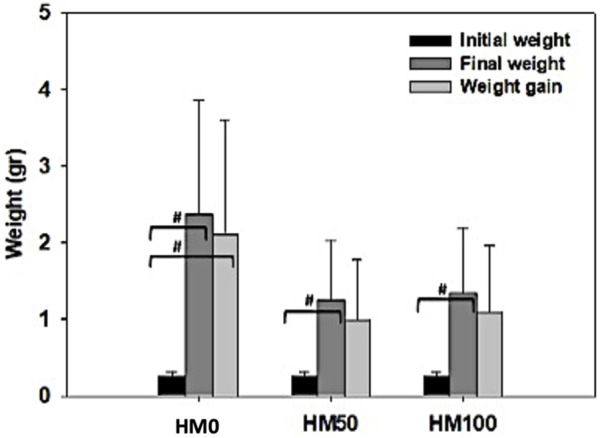
Initial, final weight, and weight gain (mean ± stedv) between different dietary treatments (HM0, HM50 and HM100) in *P. leptodactylus* juveniles. Hashtag (#) depicts statistically significant differences (*p* < 0.05) between final weight or weight gain and initial weight. HM0 represents the control group while HM50 and HM100 the groups with 50% and 100% HM inclusion respectively.

Significant differences (*p* < 0.05) were observed among the three dietary groups of crayfish at the end of the feeding trial (Table 3). Survival rate was low (12.7%–27.6%) in all dietary groups with both groups of crayfish feeding on the HM-based diets (HM50, HM100) having significantly higher survival rate compared to the control HM0 group, while the SR was similar (*p* > 0.05) between the HM50 and HM100 groups. Αt the end of the trial, a great variability of the final weight of crayfish was observed in all dietary groups with the mean final weight of the control group being significantly higher than both the HM50 and HM100 groups. The mean final weight of HM50 and HM100 were similar (*p* > 0.05). A similar trend was also observed for the mean weight gain and SGR among groups. Thus, the crayfish fed with the control diet had significantly higher values compared to the HM groups, while the HM50 and HM100 exhibited similar values. Furthermore, the HM0 group had a significantly lower FCR compared to the HM groups, while the HM50 and HM100 had a similar FCR (Figures 4, 5).

**TABLE 3:** Growth performance and feed utilization efficiency of juvenile *P. leptodactylus* fed with three different diets with different substitution levels of *H. illucens* meal.

Parameters	Treatments		
	HM0	HM50	HM100
Survival rate (SR,%)	12.7 ± 0.21	27.6 ± 0.14*	27.1 ± 0.14*^+^
Initial weight (g)	0.26 ± 0.05	0.26 ± 0.05	0.26 ± 0.05
Final weight (g)	2.37 ± 1.50	1.25 ± 0.79	1.34 ± 0.86
Weight gain (WG, g)	2.11 ± 1.50	0.99 ± 0.80	1.10 ± 0.86
Specific growth rate (SGR, %/day)	2.22 ± 0.58	1.40 ± 0.71*	1.44 ± 0.64*
Feed conversion ratio (FCR)	6.55 ± 4.18	19.04 ± 12.64*	19.30 ± 10.29*
PER	39.20 ± 22.05	16.57 ± 11.98*	16.41 ± 12.80*
Feed intake (g)	13.44 ± 7.32	16.27 ± 3.22	16.68 ± 0.93

Values represent means ± st. deviation (n = all alive crayfish). Asterisk (*) depicts statistically significant differences (*p* < 0.05) between HM, treatments and HM0, while cross (+) depicts statistically significant differences (*p* < 0.05) between HM50 and HM100 treatments.

### 3.2 Whole body composition

The whole-body composition of *P. leptodactylus* juveniles fed with the HM-based diets and the control one (HM0) are shown in Table 4. Although, the body moisture, protein, lipid and ash contents were similar (*p* > 0.05) among the three dietary groups, the diet seemed to have a statistically significant influence on the body composition of the animals. In particular, the crayfish fed with the HM-based diets had increased moisture, protein, and ash contents, and decreased lipid contents in comparison with the control group.

**TABLE 4:** Whole body composition (g/100 g fresh material) of juvenile *P. leptodactylus* individuals fed with three different diets with *H. illucens* meal substitution.

Chemical composition	Treatments		
	HM0	HM50	HM100
Moisture (%)	39.56 ± 0.05	44.91 ± 0.01*	43.41 ± 0.01*^+^
Crude protein (%)	9.62 ± 0.03	12.52 ± 0.06*	12.22 ± 0.01*^+^
Crude lipid (%)	7.83 ± 0.03	6.63 ± 0.03*	5.87 ± 0.03*^+^
Ash (%)	28.93 ± 0.11	35.10 ± 0.10*	32.22 ± 0.10*^+^

Asterisk (*) depicts statistically significant differences (*p* < 0.05) between HM, treatments and HM0, while cross (+) depicts statistically significant differences (*p* < 0.05) between HM50 and HM100 treatments. Mean value ± S.D. (n = 3).

### 3.3 Fatty acids profiles

**FIGURE 6:**
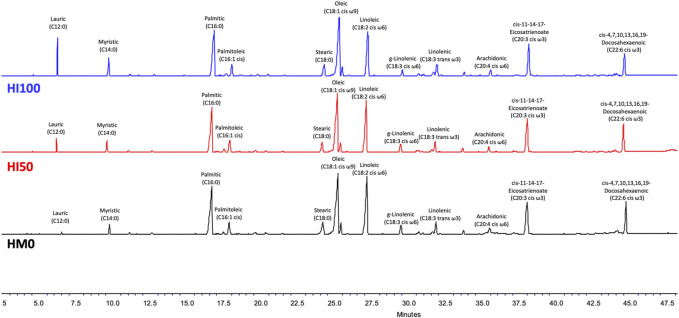
GC-FID chromatograms of the three different dietary conditions. With black color is depicted HM0 (control) condition, while red and blue color is for HΜ50 and HM100, respectively. HM0 represents the control group while HM50 and HM100 the groups with 50% and 100% HM inclusion respectively.

The fatty acid profiles of *P. leptodactylus* juveniles fed with the HM-based diets and the control diet (HM0) are shown in Table 5. Differences were observed among the three different treatments regarding the classic indices such as ∑SFAs; ∑PUFAs; ∑MUFAs. In all dietary groups, the ∑MUFAs and ∑PUFAs were found to be higher than the ∑SFAs. In all dietary groups C16:0 (palmitic acid) was the dominant SFA, C18:1 cis ω9 (oleic acid) was the dominant MUFA, while C18:2 cis ω6 (linoleic acid) represented the major PUFA. More specifically, a significant increase in the ∑SFΑ was observed, as the inclusion level of *Hermetia* meal was increased in the diet. In particular, C12:0, C14:0 and C16:0 followed the increase pattern, however C18:0 was higher (*p* < 0.05) in the HM0 group compared to HM50 and HM100 crayfish. A slight increase in the ∑MUFA was observed, as the inclusion level of *Hermetia* meal was increased in the diet but it was not statistically significant. In contrast with ∑SFA and ∑MUFA, a statistically significant decrease was observed in ∑PUFA as the inclusion level of *Hermetia* meal was increased. Among them, in C20:3 cis ω3 no significant differences were observed between the different groups while C22:6 cis ω3, C18:2 cis ω6, C18:3 cis ω6, C18:1 cis ω9 was higher (*p* < 0.05) in the HM0 group compared to HM50 and HM100 crayfish. On the other hand, C20:4 cis ω6 although found in low levels, a significant increase was observed in HM100 group in comparison with the other two group, while a significant decrease was observed in HM50 group again compared to the other two. The GC-FID chromatograms of tree different dietary conditions are depicted in the Figure 6.

**TABLE 5:** The fatty acid profile analysis (g/100 g of total fatty acids) of *P. leptodactylus* juveniles fed with three different dietary regimes (HM0, HM50, HM100).

Fatty acid	Treatment		
	HM0	HM50	HM100
Lauric (C12:0)	0.16 ± 0.00	1.32 ± 0.01*	3.78 ± 0.04*^+^
Myristic (C14:0)	1.62 ± 0.03	2.31 ± 0.01*	3.52 ± 0.01*^+^
Myristoleic (C14:1 cis-9)	0.37 ± 0.03	0.28 ± 0.01	0.42 ± 0.08^+^
Pentadecanoic (C15:0)	0.38 ± 0.04	0.36 ± 0.02	0.39 ± 0.02
Pentadecenoic (C15:1 cis-10)	0.14 ± 0.01	0.09 ± 0.01	0.17 ± 0.04^+^
Palmitic (C16:0)	17.28 ± 0.01	17.73 ± 0.08*	18.33 ± 0.04*^+^
Palmitoleic (C16:1 cis)	3.07 ± 0.00	3.43 ± 0.01*	3.39 ± 0.00*^+^
Heptadecanoic (C17:0)	0.25 ± 0.01	0.27 ± 0.00	0.36 ± 0.01*^+^
Heptadecenoic cis (C17:1 cis-10)	0.3 ± 0.07	0.26 ± 0.01	0.27 ± 0.01
Stearic (C18:0)	3.99 ± 0.24	3.26 ± 0.02*	3.68 ± 0.02^+^
Οleic (C18:1 cis ω9)	24.87 ± 0.16	26.31 ± 0.04*	25.9 ± 0.11*^+^
Linoleic (C18:2 cis ω6)	20.20 ± 0.18	18.61 ± 0.02*	14.95 ± 0.05*^+^
g-Linolenic (C18:3 cis ω6)	2.25 ± 0.01	2.14 ± 0.02*	1.64 ± 0.01*^+^
cis-11-Eicosenoic (20:1 cis ω9)	0.30 ± 0.03	0.22 ± 0.00*	0.42 ± 0.00*^+^
Linolenic (C18:3 trans ω3)	2.82 ± 0.05	2.76 ± 0.02	3.03 ± 0.02*^+^
Heneicosanoic (C21:0)	0.98 ± 0.01	1.02 ± 0.02*	0.99 ± 0.01
cis-11,14-Eicosadienoic (C20:2 cis ω6)	0.11 ± 0.00	0.02 ± 0.03*	0.09 ± 0.00^+^
Behenic (C22:0)	0.15 ± 0.03	1.34 ± 0.08*	0.11 ± 0.03^+^
cis-8,11,14-Eicosatrienoate (C20:3 cis ω6)	2.46 ± 0.05	0.14 ± 0.01*	1.71 ± 0.02*^+^
Erucic (C22:1 cis ω9)	0.17 ± 0.02	0.21 ± 0.03	0.15 ± 0.02
cis-11-14-17-Eicosatrienoate (C20:3 cis ω3)	10.56 ± 0.13	10.76 ± 0.03	10.78 ± 0.05
Arachidonic (C20:4 cis ω6)	0.16 ± 0.01	0.11 ± 0.00*	0.19 ± 0.00*^+^
cis-5,8,11,14,17-Eicosapentaenoic (C20:5 cis ω3)	0.17 ± 0.02	0.12 ± 0.03	0.16 ± 0.02
Nervonic (C24:1 cis ω9)	0.18 ± 0.04	0.14 ± 0.01	0.28 ± 0.01*^+^
cis-4,7,10,13,16,19-Docosahexaenoic (C22:6 cis ω3)	7.07 ± 0.07	6.82 ± 0.03*	5.28 ± 0.03*^+^
Saturated	24.45 ± 0.84	26.86 ± 1.32	30.63 ± 0.91*^+^
Monounsaturated	29.98 ± 0.84	30.08 ± 1.38	30.46 ± 0.95
Polyunsaturated	45.09 ± 0.94	40.37 ± 1.97*	37.18 ± 1.02*
Total ω3	20.33 ± 0.45	19.92 ± 0.98	18.91 ± 0.46
Total ω6	24.76 ± 0.50	20.46 ± 0.99*	18.26 ± 0.56*^+^
PUFA/SFA	1.85 ± 0.04	0.66 ± 0±0.00*	0.62 ± 0.01*
ω6/ω3	1.22 ± 0.00	4.9 ± 0.24*	5.48 ± 0.17*^+^
∑C18:1 ω9, C18:1 ω6, C20:4 ω6, C18:3 ω3, C20:3 ω6, C20:5 ω3, C22:6	62.61 ± 4.87	27.36 ± 1.33*	31.51 ± 0.90*
∑C14:0, C16:0	18.90 ± 0.02	3.71 ± 0.01*	3.81 ± 0.08*
Η/Η	3.31 ± 0.26	7.37 ± 0.38*	8.27 ± 0.20*^+^

Asterisk (*) depicts statistically significant differences (*p* < 0.05) between HM, treatments and HM0, while cross (+) depicts statistically significant differences (*p* < 0.05) between HM50 and HM100 treatments. Mean value ± S.D. (*n* = 3).

### 3.4 Multivariate analysis

**FIGURE 7:**
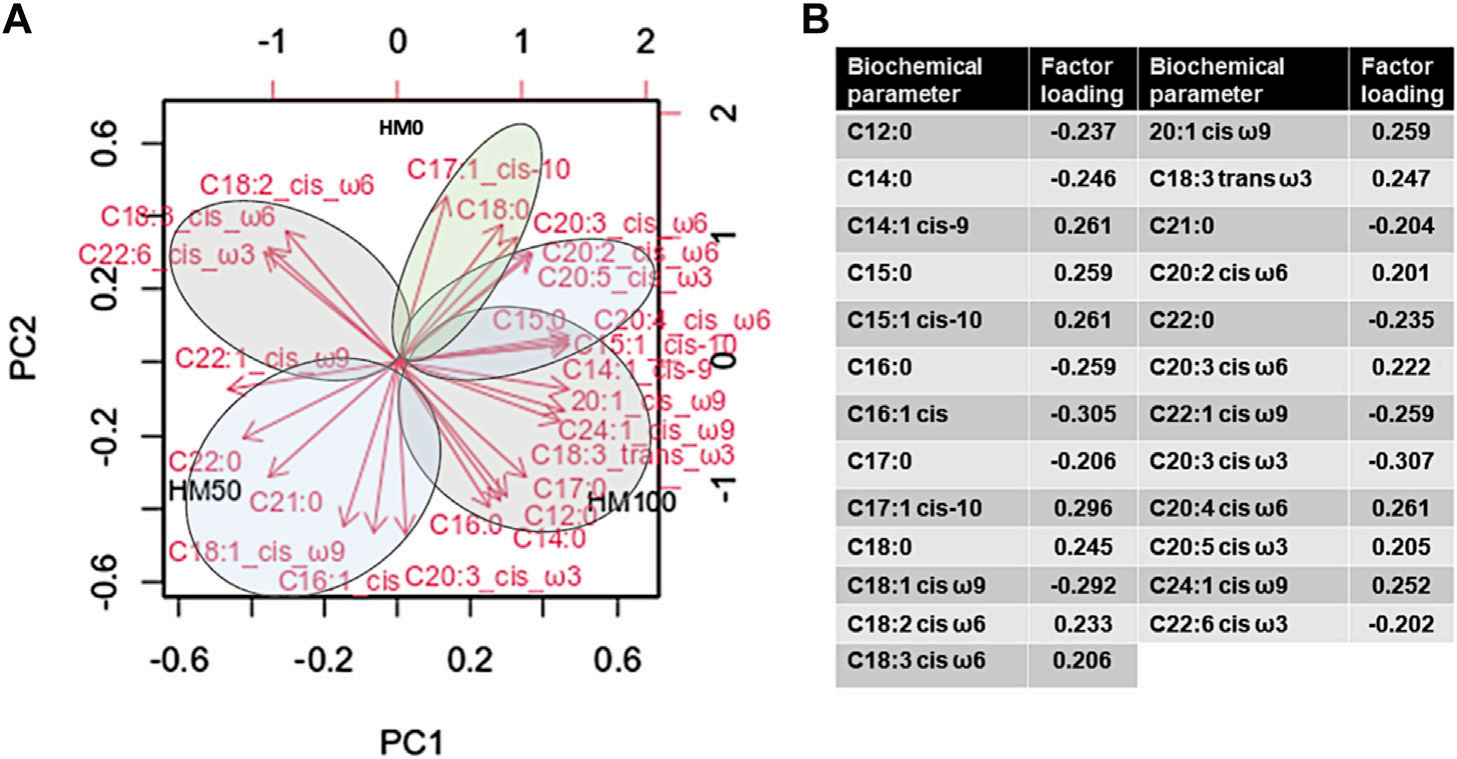
**(A)** Variable correlations with each of the first two principal components (PCs) in the multivariate analysis. The PCA was generated from the complete fatty acids dataset. Parameters with red vector arrows were included as predictors in constructing the PCA. **(B)** Analytical table of the contribution of fatty acids according to factor loadings. HM0 represents the control group while HM50 and HM100 the groups with 50% and 100% HM inclusion respectively.

The PCA analysis (principal components as extraction method) was applied to statistically define differences in the levels of fatty acids under different dietary regimes (Figure 7). PC1 explained 38.04% of the variance. Specifically, the fatty acids that were positively correlated with scores on PC1 were C14:1 cis-9 (myristoleic), C15:0 (pentadecanoic), C15:1 cis-10 (pentadecenoic), 20:1 cis ω9 (cis-11-eicosenoic), C18:3 trans ω3 (linolenic), C20:2 cis ω6 (cis-11,14-eicosadienoic), C20:4 cis ω6 (arachidonic), C20:4 cis ω6 (arachidonic) and C24:1 cis ω9 (nervonic). In contrast to the above, C22:0 (behenic), C22:1 cis ω9 (rucic) and C22:6 cis ω3 (cis-4,7,10,13,16,19-docosahexaenoic) were negatively correlated with scores on PC1. On the other hand, fatty acids positively correlated to PC2 (which explained 32.44% of the variance) were: C17:1 cis-10 (heptadecenoic cis), C18:0 (stearic), C18:2 cis ω6 (linoleic), C18:3 cis ω6 (g-linolenic) and C20:3 cis ω6 (cis-8,11,14-eicosatrienoate). In contrast, C12:0 (lauric), C14:0 (myristic), palmitic (C16:0), C16:1 cis (palmitoleic), C17:0 (heptadecanoic), C18:1 cis ω9 (oleic), C21:0 (heneicosanoic) and C20:3 cis ω3 (cis-11-14-17-eicosatrienoate) were negatively correlated to PC2. The cumulative value of PC1 and PC2 was 70.48%. Fatty acids correlating to control group form light green clusters, the ones correlating (positively or negatively) to HM50 form light green clusters, while the ones correlating to HM100 form light grey clusters.

## 4 Discussion

To the best of our knowledge, this is the first study regarding the substitution of fishmeal with *H. illucens* meal in *P. leptodactylus*. In the present study, *P. leptodactylus* juveniles were fed with three isonitrogenous, isolipidic and isoenergetic diets where the fishmeal protein of the control was replaced by 50% and 100% with *H. illucens* meal. Therefore, the effects of dietary HM inclusion were assessed on growth performance, whole body composition and fatty acid profiles of crayfish.

### 4.1 Growth performance and feed utilization

The use of *H. illucens* meal in the diet significantly affected the survival, growth performance and feed utilization of *P. leptodactylus*. It is worth mentioning that the survival rates observed in this study were similar (Ulikowski et al., 2006; Erol et al., 2017) or lower with those reported in other studies with the species (Mazlum et al., 2011; Hu et al., 2019). Certainly, the fact that *P. leptodactylus* is not yet a conventional farmed crustacean species, with rearing protocols and nutrient balanced diets still being investigated, may partly explain the low survival rates observed in the present study. In addition, the low survival may be due to the long period (88 days in total) that the juveniles were kept under laboratory conditions prior to the experiment and/or to the longer experimental period practiced (98 days in total) compared with that of Mazlum et al. (2021), which may have enormously stressed the crayfish. This can be further confirmed from the similar survival rates observed in the studies from (Ulikowski et al., 2006) and (Wang and Chen, 2005) where the juveniles’ captivity lasted for 92 and 90 days respectively. Moreover, the long period that the berried females were kept under captivity conditions may have also been a stressful factor for the progeny.

Nevertheless, the crayfish fed on the *H. illucens* diets exhibited a significantly higher survival rate than those fed on the fishmeal diet. The above observation could be attributed to the boost of the innate immune system caused by the HM consumption (Mousavi et al., 2020). Insect meals contain chitin that has been observed to regulate immune system of both fish and shrimps (Esteban et al., 2000; Wang and Chen, 2005). In addition, studies have shown that short fatty acids such as lauric acid, which is included in HI meal in the highest amount among IMs (up to 60%) (Spranghers et al., 2017; Borrelli et al., 2021) and peptides (Jozefiak and Engberg, 2017), also contained in IM, seem to exhibit antimicrobial activity and can exert a positive effect on the wellbeing of the targeted animal. A higher survival rate was observed in European seabass (*Dicentrarchus labrax*) when *H. illucens* meal included in the diet (Abdel-Latif et al., 2021) and was also observed in Pacific white shrimp after the 50% inclusion of *T. molitor* meal (Motte et al., 2019).

A great variability of the final weight of crayfish was observed in all dietary groups and this might be attributed to differences in the genetic backround of the individuals (Mills and McClaud, 1983; Gydemo et al., 1990; Curtis and Jones, 1995). Similarly, a great variability of individual growth rate has been also observed in another study with *P. leptodactylus* (Harlıoğlu, 2009) as well as in other species, such as *Astacus* (Gydemo et al., 1990), *C. quadricarinatus* (De Boulay et al., 1993; Curtis and Jones, 1995) *Austropotamobius pallipes* (Pratten, 1980). Fishmeal replacement by *H. illucens* meal significantly impaired the growth performance and feed utilization of *P. leptodactylus* at both inclusion levels. This denotes that even an inclusion level 164 g/Kg of diet (as that practiced in HM50 diet) was not suitable for the nutrition of the species and maybe a lower inclusion level is needed. The lower growth and feed efficiency was not attributed to a lower feed acceptance, as the HM-fed crayfish exhibited a similar feed intake with the control group but may be due to a lower digestibility and/or to lower dietary essential amino acids. The digestibility of HM in fish is considered to be high and close to that of fishmeal (Arango et al., 2004; Bosch et al., 2016; Gasco et al., 2022), but its high chitin content may also lower its nutrient digestibility (Kroeckel et al., 2012; Dumas et al., 2018). Regarding amino acids, HM is known as a good source of lysine, but it is limited in methionine (Makkar et al., 2014). That was the reason for supplementing the present experimental diets with methionine, while an estimation of the levels of all essential amino acids (Table 2) revealed that not major differences existed among the diets. However, as not any amino acid analysis was performed, perhaps a lower digestibility of certain amino acids could have impaired the growth of HM-fed animals. Furthermore, its remains unknown if the added amino acids have the same absorption and digestibility in comparison with those are included naturally in the diets. For carnivorous or opportunistic crayfish insects are part of their natural diet. Thus, it is reasonable to exhibit a preference on this feed type (Henry et al., 2015). From a sensory point of view, when monounsaturated to polyunsaturated fats ration is low, they lead to negative texture, odor and flavor. Thus, due to its chemical composition, BSFL may not be the optimal feed regarding nutritional value but seems to be tastier than the other insects (Wang and Shelomi, 2017). As a result, the increased feed intake observed towards when HM was included in the diet, may be attributed to better taste, rather than nutrition factors.

Unfortunately, so far there is not any other study assessing the use of *H. illucens* meal in the diet of *P. leptodactylus*. Mazlum et al. (2021) used the mealworm meal (*T. molitor*) in the diet of *P. leptodactylus* and found that the total fishmeal replacement by this IM did not impair growth and feed efficiency. The authors have also reported an even better performance of the crayfish when *T. molitor* meal replaced fishmeal at 50%.

Concerning the effects of HM in the diet of other crustacean and fish species, Wang et al. (2022) proposed that the optimum inclusion of HM in the diet of *C. quadricarinatus*, was 17.1%, probably implying that for better growth performance the substitution level with HM should be lower than 50%. Additionally, growth performance of juvenile Pacific white shrimp *L. vannamei* was similar to the control group when the fishmeal replacement with HM was restricted to less than 25% (Cummins et al., 2017) and decreased significantly with a 30% replacement (Chen et al., 2022). Significant reduction in the growth, feed intake, and protein efficiency ratio was also observed with 100% fishmeal replacement by HM in the diet of African catfish (*Clarias gariepinus*) (Adeoye et al., 2020). In addition, 25% and 30% fishmeal replacement with black soldier fly larvae meal caused a significant reduction in growth performance of juvenile yellow catfish, *Pelteobagrus fulvidraco* (Hu et al., 2017). Katya et al. (2017) reported an insignificant effect on WG and SGR of juvenile barramundi, *Lates calcarifer* when fishmeal replacement by HM was up to 50%, but growth was impaired at a higher replacement level. Karapanagiotidis et al. (2023) reported that the inclusion of a full-fat HM even at low levels (95 g/kg replacing 9% of dietary fishmeal) significantly depressed the growth performance of gilthead seabream (*Sparus aurata*), while the defatted form of the HM is more readily accepted and can be successfully included at 81–104 g/kg in the diet of this species. Similarly, Kroeckel et al. (2012) also highlighted the lower palatability of HM when included at high dietary levels that in turn negatively affected the growth of juvenile turbot (*Psetta maxima*). A reduced feed efficiency has been observed in yellow catfish feeding on HM-based diets replacing fishmeal (Xiao et al., 2018), as well as no harmful effects on both digestibility and growth performance in Japanese seabass *Lateolabrax japonicus* (Wang et al., 2019).

### 4.2 Effect of HM inclusion on body composition and fatty acid profile

Regarding the whole-body composition, it was found that this was significantly influenced with the inclusion of HM in the diet. More specifically, the moisture, protein and ash contents were lower, and lipid contents were higher in the animals fed the control diet (HM0) in comparison to the other two dietary groups (HM50 & HM100). In fact, the level of HM inclusion (HM50 vs. HM100) had also exerted a significant effect on the nutrient composition of the animals. These results denote that the HM was metabolized at a different degree in the body of *P. leptodactylus* compared to the fishmeal. This is confirmed by the lower protein efficiency ratio (PER) that was found in the HM-fed crayfish that in turn may has led to a higher protein retention in the body instead of catabolizing this to growth. Furthermore, the whole-body composition analysis revealed that the crude lipid content was decreased by the increase of HM level in the crayfish diet. It is possible this to be related with the high chitin levels of HM that are known to reduce the lipid absorption in the gastrointestinal tract of aquatic organisms (Tanaka et al., 1997) and turn to result in lower in a lower lipid body deposition. In line with the present study, dietary inclusion of IMs (*Spodoptera littoralis* and *H. illucens*) were found to decrease the carcass lipid content in Nile Tilapia (Muin et al., 2017; Amer et al., 2021). Furthermore, the moisture content decrease followed the increase in lipids. This relation type has been studied and has been observed by many previous researchers in fish (Ahmed et al., 2022). More specifically, the whole-body moisture content seemed to be inversely dependent to whole body lipid content as it increases or decreases as lipids are utilized or stored (Shearer, 1994). The higher ash contents observed in the bodies of HM-fed crayfish cannot be easily interpreted as the HM (Table 1) and HM-based diets (Table 2) contained lower ash levels compared to fishmeal diets. Certainly, a higher ash content imply a higher mineral content, and this can be associated with the processes of exoskeleton formation. In this life stage, it is expected that juveniles undergo multiple ecdysis, which indicating a fine welfare status (Aiken and Waddy, 1992). Furthermore, the analysis included the whole body, which means that exoskeleton was included, probably resulting in increased ash content. It is worth mentioning that Mazlum et al. (Mazlum et al., 2021) using dietary *T. molitor* did not find significant alteration of the ash content of *P. leptodactylus* juveniles.

Additionally, the fatty acid compositions of the three dietary groups (HM0, H50, H100) were evaluated. A statistically significant increase in the C12:0 (lauric acid) content was observed along with the concomitant increase in HM meal substitution in the diet. This observation could be explained by the fatty acids composition of the HM diet that was found to be rich in C12:0 (lauric acid), C18:1 cis ω9 (oleic acid) and C18:2 cis ω6 (linoleic acid), but poor in C20:4 cis ω6 (arachidonic acid), C22:6 cis ω3 (cis-4,7,10,13,16,19-Docosahexaenoic) and C20:5 cis ω3 (cis-5,8,11,14,17-Eicosapentaenoic) content in comparison to the control fishmeal (Esteban et al., 2000). Furthermore, an increase in the ∑SFAs combined with a decrease in ∑PUFAs and ∑ω6 fatty acids were observed following the increase in HM meal substitution in the diet. However, no statistically significant differences were observed in ∑MUFAs and ∑ω3 fatty acids content. The PCA analysis in the present study, confirmed with the cluster formation, the fact that the three different dietary conditions, influenced the fatty acid profile of the animals. From earlier (St-Hilaire et al., 2007; Sealey et al., 2011) to latter studies (Borgogno et al., 2017; Belghit et al., 2018; Belghit et al., 2019) in Atlantic salmon and rainbow trout it is proposed that the partial or overall substitution of fishmeal with HM meal may increase ∑SFAs, while may decrease the ∑PUFAs content. The above observation could be attributed to the higher HM meal content in C12:0 (lauric acid), and as a result a subsequent fillet increase in ∑SFAs was expected in fish fed HM diet (Zarantoniello et al., 2022). Elevated lauric acid levels and a decrease in ∑PUFAs was also found in the whole fish of zebrafish (*Danio rerio*) after a feeding trial with black soldier fly prepupae (Zarantoniello et al., 2018).

The demand for seafood production is under continuous increase, a trend which is also followed by the fishmeal production (FAO, 2016). It is therefore considered that fishmeal is no longer sustainable (Tacon and Metian, 2008). Therefore, the scientific community searches for potential alternative protein sources for fishmeal substitution. Among them, plant protein sources such as soybean meal (SBM), rapeseed meal (RSM), groundnut oil cake (GNC), cottonseed meal (CSM) and sunflower oil cake (SFC) are good representatives (Jannathulla et al., 2019). SBM is probably the best alternative plant protein source mainly due to its high protein content and to its balanced amino acid profile. However, its high demand led to an increase in its cost and a decrease in its availability (Tacon et al., 2011). As a result, the interest towards the other plant protein sources (RSM, GNC, CSM and SFC) is increasing due to their wide availability, desirable nutrient profile and their low cost (Jannathulla et al., 2019). However, in organisms feeding on animal protein as well the complete fishmeal substitution with plant protein sources seems inevitable as it is associated with reduced overall performance and health status (Oliva-Teles et al., 2015). Thus, the use of other protein source alternatives in aquaculture feeds such as poultry by-product meal (PBM); blood meal (BM) and meat and bone meal (MBM) increased (Gasco et al., 2018). All the above terrestrial processed animal proteins (PAP) are characterized by a high content in useful amino acids such as lysine, histidine, sulphur amino acids, and arginine plus they are low-cost alternatives (Goda et al., 2007). However, there are some inhibitory factors towards their wide use, such as low digestibility, absence of some essential amino acids (Gasco et al., 2018) and the prohibition of their use in the EU. Another one potential solution towards sustainability are the amphipods belonging to *Gammarus* genus. Studies showed that they can substitute fishmeal at levels of 10%–20% without any adverse effects on growth performance and survival rate of fish. However, *Gammarus* meal cost is higher in comparison to fishmeal and this phenomenon is mainly attributed to the absence of a developed culture method (Harlıoğlu and Farhadi, 2018). Although insect meals are gaining more and more attention there are some obstacles towards their use in aquaculture feeds. They are characterized by increased cost when compared to conventional protein sources (Niyonsaba et al., 2021) and they are characterized by high chitin content which lower their digestibility (Gasco et al., 2016). Additionally, some deficiencies in essential amino acids have been reported (Henry et al., 2015). Although nutritional value of insect meals are directly associated with the substrate and the treatment used to culture them, their protein content seem to be relatively stable (Henry et al., 2015). Furthermore, IM found to boost fish immune system resulting iin improved overall health performance (Mousavi et al., 2020).

To conclude, here we found that the dietary fishmeal replacement by *H. illucens* meal either partially (at 50%) or totally, negatively affected the growth performance and feed utilization of *P. leptodactylus*, although improved the survival. In addition, the HM inclusion in the diet significantly altered the whole-body chemical composition of the crayfish signifying a different metabolic utilization compared to fishmeal. Furthermore, the inclusion of dietary HM significantly reduced the contents of ∑SFAs, ∑PUFAs and ∑ω6 fatty acids, as well as those of C22:6 cis ω3 and increased the ω6/ω3 and Η/Η ratios in the body. In parallel with improvements in balanced diets and in culture conditions, further studies are also necessary on the use of lower HM dietary levels and with other insect meals in order to enlighten the suitability of insect meals in the nutrition of *P. leptodactylus*.

## 5 Conclusion

The present study provides a substantial contribution for future experiments regarding the establishment of a standard diet composition for the development of narrow clawed crayfish rearing protocol, as so far existing data are extremely limited and contradictory. Further, our data support the welfare and good performance of freshwater crayfish *P. leptodactylus* under captivity. Fishmeal substitution with insect meals and more specifically with *H. illucens* mealworm meal is still under investigation. It seems that each organism should be evaluated independently as the nutritional needs of each one differs. Another key point is the determination of the optimal inclusion level of IM. The above, is of major importance both for environmental issues and for production cost reduction. However, when the replacement amount exceeds this optimum level, the growth performance (i.e., in the present study) and physiological conditions (Chen et al., 2022) of the organism may be negatively affected. Here, we found that SR exhibit a statistically significant increase in the diet groups with ΗΜ inclusion, while the SGR, FCR and WG were decreased affected again statistically significant. Furthermore, regarding the whole-body chemical composition not big differences in absolute values were observed however were statistically significant. Lastly, from the fatty acid profile analysis was observed that ∑SFAs, ∑PUFAs and ∑ω6 fatty acids were statistically significant reduced following the ΗΜ substitution. Finally, to our knowledge this is the first study evaluating the HM inclusion into freshwater crayfish diet, a promising insect of high importance in animals’ nutrition.

## Data Availability

The raw data supporting the conclusion of this article will be made available by the authors, without undue reservation.
